# Assessing the thermal-moisture functional performance of two sets of work uniform by S-smart simulation

**DOI:** 10.1186/2046-7648-4-S1-A90

**Published:** 2015-09-14

**Authors:** Wenfang Song, Albert PC Chan, Yueping Guo, Yang Yang, Faming Wang

**Affiliations:** 1 Laboratory for Clothing Physiology and Ergonomics (LCPE), the National Engineering Laboratory for Modern Silk, Soochow University, Suzhou, China; 2 Department of Building and Real Estate, The Hong Kong Polytechnic University, Kowloon, Hum Hon, Hong Kong

## Introduction

Construction workers are susceptible to heat stress in summer of Hong Kong. Wearing work uniform with good thermal-moisture functional performance (TMFP) is considered as one of the effective measures to protect workers from heat stress. However, there is a lack of scientific research to design workers' uniform based on heat-moisture engineering. This study aims to predict the TMFP of the selected fabrics by a S-smart system [1] under a stressful thermal environment.

## Methods

The fabric characteristics of the examined knitted T-shirts (T1 and T2) and woven full-length pants (P1 and P2) were listed in Table 1. The S-smart system was adopted to simulate TMFP of clothing with the input parameters listed in Table 1 (fabric characteristics) and Table 2 (body characteristics, activities and environmental condition) [2]. The simulation results were the human physiological responses, including the mean skin temperature (*T*_sk_) and core temperature (*T*_c_).

**Table 1:** Fabric characteristics T-shirt fabrics (T1 and T2) and full-length pants (P1 and P2).

Fabrics	Fabric content	Weight (g.m^-2^)	Thickness (mm)	OMMC	AR (KPa.s.m^-1^)	WVP (g.m^-2^.h^-1^)	k (w.m^-1o^C^-1^)
T1	100% cotton	134.37	0.55	0.66	0.24	657.8	0.06
T2	100% Coolmax^®^	146.40	0.62	0.8	0.06	593.1	0.06
P1	60% cotton blended with 40% polyester	174.95	0.35	0.69	1.58	496	0.05
P2	100% cotton with Dry-Inside^® ^technology	185.26	0.48	0.86	1.96	530.6	0.06

Note: OMMC-overall moisture management capacity; AR-air resistance; WVP-Water vapour permeability; k-heat conductivity

**Table 2:** The design case in computer: body characteristics, activities and environmental condition.

	The design case in computer simulation
Body characteristics	65.6 kg and 168.5 cm
Activity and environmental condition	Seated relaxed (58 w.m^-2^), and (30°C, 50 %RH)-Standing activity (163 w.m^-2^), and (30.7°C, 65 %RH)- Standing activity (248 w.m^-2^), and (33°C, 54 %RH)-moving activity (229 w.m^-2^), and (34°C, 42 %RH)-moving activity (167 w/m^2^), and (32°C, 41 %RH)-recovery (58 w.m^-2^), and (31°C, 44 %RH)

## Results and discussion

**Figure 1:**
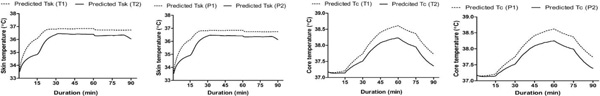
**Comparison of core and skin and core temperature changes among T-shirts (T1 and T2) and pants (P1 and P2)**.

The computer simulation in Figure 1 showed that T2 and P2 had better thermal-moisture performance in terms of predicted core and skin temperatures than T1 and P1, respectively. However, only a marginal difference was presented. This is might due to the excellent OMMC and lower AR of T2 and P2 compared to T1 and P1, promoting evaporative heat loss more efficiently.

## Conclusion

The computer simulation results indicated that T2 and P2 can improve thermoregulation by decreasing the predicted core and skin temperatures and microclimate humidity as compared to T1 and P1, respectively.

## References

[B1] Guo YP Heat and mass transfer of adult incontinence briefs in computational simulations and objective measurements International Journal of Heat and Mass Transfer 2013 64 133 144

[B2] Chan APC Evaluating a newly designed construction work uniform on heat stress Textile Research Journal 2015 unpublished paper

